# Bacterial biophotons as non‐local information carriers: Species‐specific spectral characteristics of a stress response

**DOI:** 10.1002/mbo3.761

**Published:** 2018-10-31

**Authors:** Lucas W. E. Tessaro, Blake T. Dotta, Michael A. Persinger

**Affiliations:** ^1^ Behavioural Neuroscience Program Laurentian University Sudbury Ontario Canada; ^2^ Department of Psychology Laurentian University Sudbury Ontario Canada; ^3^ Interdisciplinary Human Studies Laurentian University Sudbury Ontario Canada

**Keywords:** Biophotons, cell stress, *E. coli*, non‐local communication, *S. marcescens*

## Abstract

Studies by Alexander Gurwitsch in the 1920' s with onion root cells revealed the phenomenon of *mitogenetic radiation*. Subsequent works by Popp, Van Wijk, Quickenden, Tillbury, and Trushin have demonstrated a link between Gurwitsch's mitogenetic radiation and the biophoton, emissions of light correlated with biological processes. The present study seeks to expand upon these and other works to explore whether biophoton emissions of bacterial cultures is used as an information carrier of environmental stress. Bacterial cultures (*Escherichia coli* and *Serratia marcescens*) were incubated for 24 hr in 5 ml of nutrient broth to stationary phase and cell densities of ~10^7^ cells/mL. Cultures of *E. coli* were placed upon a photomultiplier tube housed within a dark box. A second bacterial culture, either *E. coli* or *S. marcescens*, was placed in an identical dark box at a distance of 5 m and received injections of hydrogen peroxide. Spectral analyses revealed significant differences in peak frequencies of 7.2, 10.1, and 24.9 Hz in the amplitude modulation of the emitted biophoton signal with respect to whether a peroxide injection occurred or not, and whether the species receiving the injection was *E. coli* or *S. marcescens*. These and the subsequent results of discriminant functions suggest that bacteria may release biophotons as a non‐local communication system in response to stress, and that these biophotons are species specific.

## 1 INTRODUCTION

The concept of non‐locality implies that appropriate stimulation of phenomena in one locus can be reflected in the phenomena at a second locus without the obvious involvement of classical mechanisms or processes of transmission such as electromagnetic fields. Dotta and Persinger first demonstrated that photon emissions from two bioluminescent reactions (hydrogen peroxide and hypochlorite) separated by 5 m to over 1 km behaved as if the two spaces were superimposed (Dotta & Persinger, 2012). There was a doubling of the photon emissions. The doubling only occurred if both reactions shared the same circularly rotating magnetic fields with varying angular velocities and specific temporal parameters. This doubling did not occur if other magnetic configurations were applied or if the injections in the two loci were not simultaneous. To discern whether there were geometries with specific parameters that might simulate these conditions, we examined the potential non‐locality of photon emissions in pairs of plates of bacteria. According to our calculations, the potentially rotating magnetic fields within circular arrays of RNA within bacteria exhibit the properties that might simulate the experimental conditions that produced the doubling of photon emissions (Dotta & Persinger, 2012). If our hypothesis was valid than injection of hydrogen peroxide into bacteria within one plate contained within a black shielded volume should be associated with changes in photon emission characteristics in a second plate housed within a black shield volume when separated by about 5 m. This would imply that "information" between bacteria separated by non‐traditional distances and involving "non‐local" processes might occur.

The human microbiome is quickly becoming a key research area particularly as evidence‐based medicine incorporates more individualized treatment schemas; however, studies depicting the impact of microbial populations on host physiology suggest there is still much work to be done to complete the picture (Sender, Fuchs, & Milo, 2016; Zmora, Zeevi, Korem, Segal, & Elinav, 2016). The number of human cells in the body is estimated to be around 10^13^ (Bianconi et al., 2013), leading to striking ratios of up to 10:1 for bacteria‐to‐human cells, implying that a human being is an organism in which 90% of its cell population is foreign. The ratio of bacterial‐host cell populations is even more important in light of the aforementioned upsurge in research articles demonstrating biochemical reactions between various species, and is by no means limited to human–bacterial interactions. Nor is it unidirectional, with recent work indicating the host contributes to gut microbiota composition as mediated through interactions with the enteric nervous system (Rolig et al., 2017).

Providing a brief albeit necessary vignette of the impact of microbiota on the host, Schwarzer & colleagues showed that the strain *Lactobacillus plantarum* was essential in maintaining proper weight gain in infant mice regardless of whether they had a nutritious or deficient diet, an effect found in humans as well (Pennisi, 2016; Schwarzer et al., 2016). These host–bacterium interactions relate directly to immune response. One study demonstrated that there was an overgrowth in *Enterobacteriaceae* populations 24 hr after severe epithelial burns, having significant impact on patient recovery and outcome (Earley et al., 2015). Furthermore, evidence now exists indicating that some forms of cancer may also be linked to changes in local microbiota, opening a new window on cancer biology (Urbaniak et al., 2016). This, coupled with the growing evidence of a bidirectional communication system between hosts and their bacterial tenants, places more importance on the mechanisms behind these interactions (Sandrini, Aldriwesh, Alruways, & Freestone, 2015).

While it is likely that traditional biochemical communication systems remain at the heart of any inter‐cellular communication complex, comparatively less research exists on non‐local communication (*i.e.,* communication occurring in absentia a classical medium). That is not to say the field is barren—on the contrary, research into such non‐local methods dates back to at least to the 1920' s with Alexander Gurwitsch and his observations on the factors contributing to cellular division (Gurwitsch, 1923). In particular, when observing dividing onion root cells he noted that they proceeded through the various stages faster when there were other cells nearby also undergoing division, and postulated a form of electromagnetic communication since termed *mitogenetic radiation* (Gurwitsch & Gurwitsch, 1926). Rephrased in modern circles as ultraweak photon emission (UPE) or biophotons are generally in the emission range of 100—1,000 photons·cm^−2^ of a given biological tissue (Popp, 1979), and are typically isolated to the ultraviolet (UV) and infrared (IR) regions of the electromagnetic spectrum (Dotta, 2014).

In the decades since, copious studies have demonstrated the existence of photons originating from biological sources. Quickenden & Que Hee showed differential levels of UPEs are tied to the cell stages of growing yeast cultures (Quickenden & Hee, 1976). Although further attempts to definitively show mitogenetic radiation with yeast were not successful, subsequent studies have revealed distinct spectral characteristics of the UPEs recorded from growing *Escherichia coli* cultures (Tilbury & Quickenden, 1988). Further along the phylogenetic tree, Kobayashi and colleagues showed the emission of a consistent 10^−12^ W·m^2^ from the exposed cortices of rats (Kobayashi, Takeda, Ito, Kato, & Inaba, 1999), within an order of magnitude of the 10^−11^ W·m^2^ recorded by Isojima *et al*. from prepared hippocampal slices (Isojima, Isoshima, Nagai, Kikuchi, & Nakagawa, 1995).

In recent years, studies exploring various aspects of UPEs have been the focus of our laboratory, and in particular have demonstrated the involvement of biophotons across numerous processes. For example, increased biophoton emissions occur when engaging in visual imagery in the order of 10^‐11^ W·m^2^ (Dotta & Persinger, 2011; Dotta, Saroka, & Persinger, 2012). Biophoton emissions are increased in one cell culture when another cell culture received light flashes, also in the order of 10^−11^ W·m^2^ (Dotta, Buckner, Lafrenie, & Persinger, 2011). The coupling of photon emissions in cells can be induced through exposure to the same electromagnetic field stimulation, where the effect is maximized again in the order of 10^−11^ W·m^2^ (Dotta, Lafrenie, Karbowski, & Persinger, 2014). Finally, the dynamic state of protein kinase A has also been linked strongly with biophoton activity (Dotta, Buckner, & Lafrenie, 2016). The importance of these latter works is the implication that the emissions of photons from biological tissues are not merely metabolic by‐products, as might be inferred from the IR spectrum, but are actual forms of biocommunication and are actively involved in cellular processes.

Some of these studies on biophoton communications have required the timed paired exposure to an identical electromagnetic field; otherwise, no effect occurred. Although still a form of non‐local communication, potentially related to Gurwitsch's mitogenetic radiation, studies requiring the incorporation of these fields will always be subject to criticism. Thus, the present study aims to demonstrate the communication between microorganisms non‐locally absent any external field presentations to investigate Gurwitsch's hypothesis. This would not be the first work to attempt to do so. Early studies by Nikolaev showed that the growth of one microbial species in a glass flask could stimulate the growth of another species in a glass flask housed within the first, although the study has received serious criticism (Nikolaev, 2000). Trushin, however, has conducted an excellent review of the probable mechanisms by which light‐mediated electromagnetic communication may occur (Trushin, 2003, 2004 ). We present here further evidence of the ability for bacterial species *Escherichia coli* and *Serratia marcescens* to communicate in absentia a classical medium as evidenced through biophoton emission spectra.

## 2 MATERIALS AND METHODS

### 2.1 Bacterial cultures

Bacterial cultures were obtained from the American Type Culture Collection (ATCC), Manassas, VA, USA. Two bacterial strains were acquired and cultured for use in the experiment: 1) *Escherichia coli* (11303), and 2) *Serratia marcescens* (pigmenting, 264). Both of these species are rod‐shaped gram‐negative facultative anaerobes found within the same genetic family of *Enterobactericeae*. These species were selected as *E. coli* is somewhat ubiquitous in terrestrial environments and has had its genome fully sequenced, whereas *S. marcescens* is an opportunistic pathogen found within the human microbiome and often being the causal agent of catheter‐related urinary tract infections; thus, both of these species routinely interact with *Homo sapiens*.

The two strains were first cultured on nutrient agar, prepared by combining the appropriate mixtures of nutrient broth powder (Oxoid CM00001) and agar powder (Fischer A360–500), both of which were obtained through Fischer Scientific (Mississauga, ON, Canada). The agar cultures, after confirmation of species, were then cultured in individual 10 ml test‐tubes housing 5 ml of nutrient broth (Oxoid CM00001). Temporally, fresh agar cultures were maintained weekly throughout the experiment, and the sub‐cultured broth samples were inoculated 24 hr prior to exposure on the photomultiplier tube. This was done so that culture density would be constant within an order of magnitude. Culture densities were 2.8 x 10^7^ CFU/mL and 1.0 x 10^7^ CFU/ml for *E. coli* and *S. marcescens*, respectively. Bacterial cultures were maintained at 37°C, with the exception of during the actual running of experiment when cultures were exposed to ambient (25°C) temperatures. Bacterial cultures to be used in the experiment were cultured in a 5 cm uncoated cell culture dish for 24 hr *in lieu* of 10 ml test tubes.

### 2.2 Photomultiplier tube assay

Observations of biophoton emissions were made by placing the bacterial cultures on the 3‐cm^2^ aperture of a Sens‐Tech, Ltd. Model DM0090C Digital Photomultiplier Tube (PMT). Measurements consisted of 3,000 samples at a rate of one sample per 20 msec (50 Hz). According to specifications, the PMT is sensitive to photons of wavelengths between 280 and 850 nm. The subject bacterial culture within the 5 cm dish was placed upon the PMT aperture, and the whole apparatus (PMT +bacteria) was placed within a 15 x 15 x 15 cm dark box with an open top to permit access. The open top was later sealed with a number of heavy black towels. The same process was conducted for the bacterial species receiving H_2_O_2_ injections, to be described shortly. Experiments were conducted in an “8 + 1” design, where prior to placing the bacterial culture upon the PMT an environmental baseline recording was made, where *baseline* is defined as a background photon emission recording. The culture was then placed on the PMT and a *run*, consisting of eight observational recordings, began.

The bacteria were observed for UPE during the eight recordings, four of which were baseline and four of which coincided with injections of hydrogen peroxide (H_2_O_2_) in a γ‐A‐γ‐A‐γ‐B‐γ‐B design. In this design, *A* represents an injection volume of 0.1 cc, *B* represents an injection volume of 0.2 cc, and *γ* denotes a baseline recording. By virtue of this design, only the first *γ* is a “true” baseline, which we accounted for in our statistical analyses, as well as potential cumulative effects of the H_2_O_2_. The injections were made via 1 mm diameter serological tubing suspended over the injection culture within the second dark box, into which the H_2_O_2_ was sent using a 5 ml laboratory syringe.

To summarize, the standard experimental run consisted of a bacterial culture being placed upon the PMT inside one dark box, a second bacterial culture was placed in a separate dark box ~5 m away and received H_2_O_2_ injections via a syringe. Injections occurred approximately 5 s into each injection run. For each run, a new plate of bacteria was used at both the PMT recording and injection sites, were again a *run* refers to the “8 + 1” design.

### 2.3 Additional experimental manipulations

Rather than simply placing one culture on a PMT and injecting peroxide into a second independent culture, we also included experimental manipulations to demonstrate the efficacy of potential non‐local communication. As mentioned previously, we originally included two peroxide injection volumes, 0.1 and 0.2 cc., and so throughout the course of the experiment we balanced the exposure paradigm to include AABB, BBAA, ABAB, and BABA (with appropriate γ). We also included a *Plate* condition, where the bacterial plate upon the PMT was either kept the same or changed to a new plate, as well as a *Box* condition where the injection plate was housed in the same dark box as the recording plate, or in a separate box ~5 m away (standard paradigm). Perhaps the most important variation was the species itself—some runs were conducted with *E. coli* as both the PMT and injection species, and some runs utilized *S. marcescens* as the injection species. All these factors were accounted for in the statistical analyses.

### 2.4 Statistical analyses

Data were analyzed using IBM Statistical Product and Service Solutions (SPSS) software (IBM Corp., Released 2016, IBM SPSS Statistics for Windows, Version 24.0. Armonk, NY: IBM Corp.). Raw PMT values were processed by first standardizing the values for a given run, then conducting a windowless spectral analysis on the standardized values. The resulting spectral analysis values were standardized and then imported into an SPSS database for further analyses. A factor analysis to reduce the number of spectral variables from 1,500 to 260 components was conducted using 570 iterations of a varimax rotation matrix whose components were extracted using principle component methods.

In addition to the standardized spectral values, the total average photon emission for each recording, as well as the average emission during 0–5, 5–10, 10–15, and 15–60 s windows were calculated and added to the dataset. To account for the observed general increase in PMT values over time, the average photon counts were first detrended. This was accomplished by using the *Curve Fit* function in SPSS to determine what regression model best fit the data. Afterward, this best fit regression was run with time as the independent variable and the resulting residuals were saved as a new variable. Additional statistical analyses as they pertain directly to results are described below.

## 3 RESULTS

### 3.1 Species‐dependent frequency response

**Figure 1:**
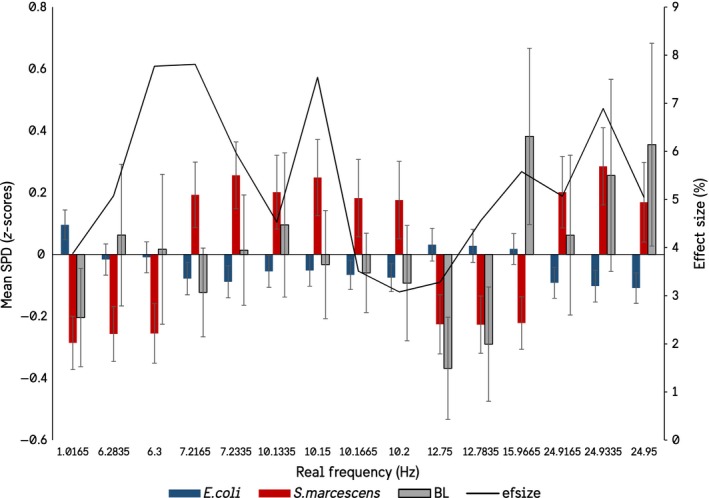
Real frequencies to which significant factor loadings can be attributed. The figure shows the mean *z*‐scored spectral power densities of the recorded *E. coli* culture when either E. coli (blue) or *S. marcescens* (red) received injections of H_2_O_2_. Baseline measures (gray) are also presented. The effect size from the ANOVA is reported as well. (SEMs)

The first objective was to determine whether there was a differential response in the observed PMT recordings resulting from changing the species receiving the H_2_O_2_ injections. The 1,500 frequency variables generated from the spectral analysis ranged from 0–25 Hz, or ½ the recording frequency of 50 Hz (20 msec) as imposed by the Nyquist Limit. Rather than investigate each of the 1,500 variables independently, first 1 Hz frequency BINs were generated using factor analyses. The resulting 260 extracted factors were then used as independent variables in an Analysis of Variance (ANOVA), where *Species Receiving H_2_O_2_ Injection* was the dependent variable. Those factors that showed a significant difference in spectral power density between the species were then matched with their respective real frequency loadings, which are summarized in Figure 1, with true baseline spectral power densities included as a comparator.

**Figure 2:**
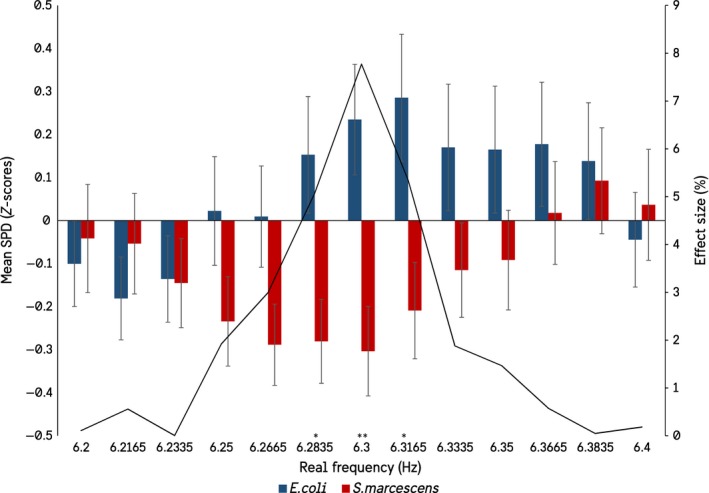
Results of the expanded ANOVA centered on the significant 6.3 Hz frequency. A rise toward significance and effect size can be seen approaching 6.3, and falling out moving away from 6.3 (SEMs)

**Figure 3:**
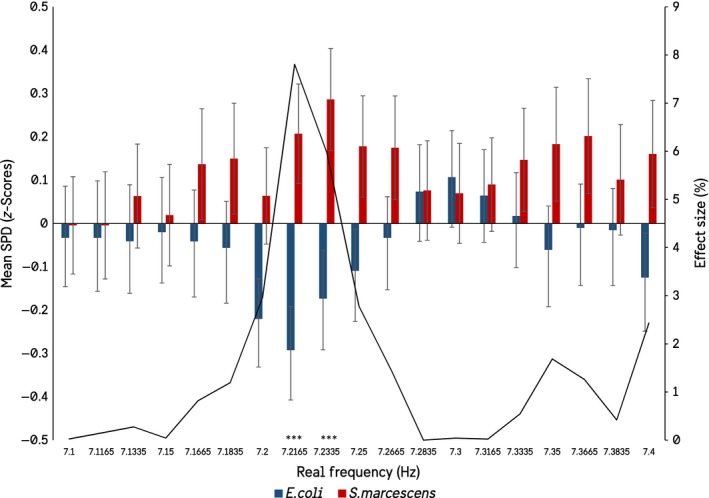
Results of the expanded ANOVA centered on the significant frequencies in the 7.1 to 7.4 Hz bandwidth (SEMs)

**Figure 4:**
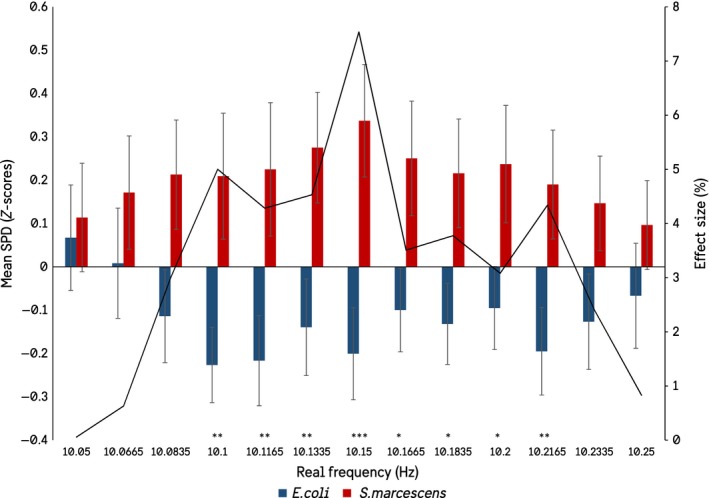
Results of the expanded ANOVA centered on the significant frequencies in the 10.05 to 10.25 Hz bandwidth (SEMs)

**Figure 5:**
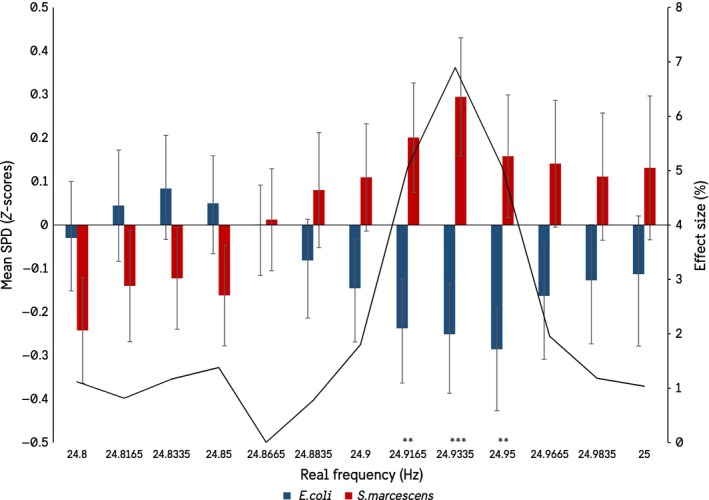
Results of the expanded ANOVA centered on the significant frequencies in the 24.8 to 25.0 Hz bandwidth (SEMs)

It seemed unlikely to us that biogenic photon effects could be isolated so punctate around particular frequencies, even accepting the summations of the statistical software. Therefore, additional analyses were conducted within a ± 1 Hz window around each of the significant frequencies in Figure 1. For example, if the target frequency is 6.3 Hz, a secondary ANOVA was conducted on the spectral power densities between 6.2 and 6.5 Hz. This resulted in a widening of the frequency windows in which significant differences in the spectral power densities between the species could be observed, as well as a loss of the 1.0165 Hz and 12–15 Hz peaks. The results show that when the significant peaks are investigated at a higher resolution there is in fact a gradation toward significance, as would be expected (Figures 2, 3, 4, 5).

In summation, 16 frequencies remained significantly different between the two bacterial species when investigating spectral power density differences: 6.2835, 6.3, 6.3165, 7.2165, 7.2335, 10.1, 10.1165, 10.1335, 10.15, 10.1665, 10.1835, 10.2, 10.2165, 24.9165, 24.9335, and 24.95 Hz.

### 3.2 Condition‐dependent changes in photon emissions

As mentioned in the *Methods*, raw averages of photon emissions for each of the individual recordings were collected as well as averages for 0–5, 5–10, 10–15, and 15–60 s windows. We reasoned that injections occurred during the 5–10 s window, thus comparisons immediately before and after the event might prove to be sources of variance. One‐way ANOVA of the four photon bins plus the run average as dependent variables with the species receiving injection as the independent variable revealed no significant differences in the average photon emissions (*p *> 0.05). Similarly, no significant differences in average photon emissions recorded were found when exploring any of the variations of the H_2_O_2_ manipulation in similar one‐way ANOVA (*p *> 0.05).

**Figure 6:**
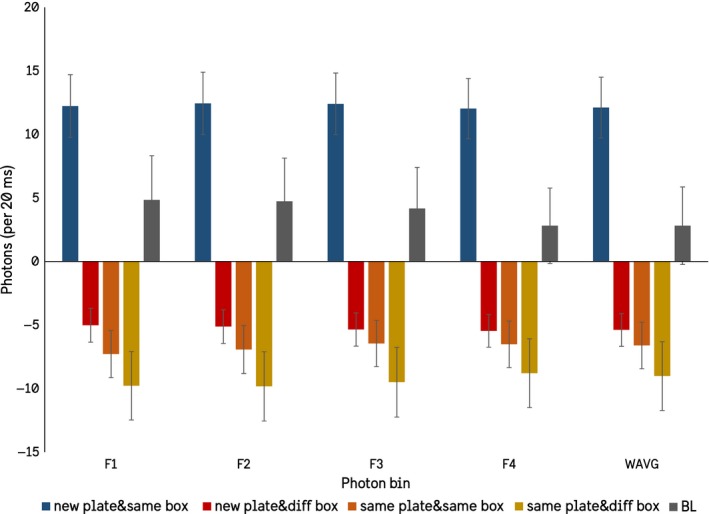
The detrended average photon counts for each of the computed windows and the overall run average presented with comparative baselines (SEMs).

A new variable was computed generating four new groups: *New PMT Plate & Same Injection Box*,* New PMT Plate & Different Injection Box*,* Same PMT Plate & Same Injection Box,* and *Same PMT Plate & Different Injection Box*. This new variable was used as the independent variable in a subsequent ANOVA with the detrended photon averages as the dependent variables, which were significant for all windows (Figure 6). Tukey's *post hoc* analyses with a critical value of 0.05 were conducted and confirm that the group *New PMT Plate & Same Injection Box* as being the greatest source of variance, having higher detrended photon counts than either *baseline* or the other three conditions, which were significantly different from *baseline* but not from each other (Figure 6).

### 3.3 Classification of condition, species using discriminant functions

Using the 260 component frequencies, a series of discriminant analyses were conducted to ascertain the nature of the biophoton effect. The first discriminant with max steps set to 5 attempted to classify “whether an injection of hydrogen peroxide occurred or not”; a function incorporating components belonging to the 1–2, 3–4, 15–17, and 22–23 Hz bandwidths produced a function with a cross‐validated classification accuracy of 69.9%, explaining only 19% of the variance (Wilks’ Λ = 0.810). A function including 17 steps was required to explain more than 50% of the variance, maximizing at 85% of variance explained with a 58‐step function and an accuracy of 92%. An identical discriminant function classifying the three hydrogen peroxide conditions (baseline (0 cc), 0.1 cc, 0.2 cc) resulted in very poor (<50%) classification accuracies.

A second discriminant analysis was conducted to classify “which bacterial species received the injection of hydrogen peroxide.” The resultant function was able to accurately classify 76% of cases in a cross‐validated, 5‐step model explaining 21% of the variance (Wilks’ Λ = 0.79). Components of the 0–1, 9–10, 12–13, 22–23, and 24–25 Hz bandwidths entered as variables. A 37 variable model was able to accurately classify 94% of cases in a cross‐validated model, achieving a maximum variance explained of 73%. Similarly, the function achieved 50% variance explained after incorporation of the 17th variable.

## 4 DISCUSSION

There is an important methodological note to consider when reviewing the results of the present study—the bacterial species receiving the hydrogen peroxide injections *was not recorded for photon emissions*; stated alternatively, we recorded the biophoton emissions from a bacterial culture that was not manipulated in any way. The null hypothesis demands that there be absolutely no differences between any baseline recordings and our “experimental condition” recordings. Yet despite this assumption, we find the opposite. When recording a culture of *E. coli* for biophoton emissions we find, unequivocally, that the culture responds reliably when another culture of bacteria in the vicinity is insulted with hydrogen peroxide. Furthermore, we find that we can discern whether the species of bacteria receiving the hydrogen peroxide is of the same species that we are recording biophotons from, or not. The results of the present study add to the already overwhelming evidence suggestive of non‐local communication; that is, communication in absentia a classical medium.

Although the results of the discriminant functions are relatively weak—explaining only 20% variance with an accuracy of 70%—the variables entering the function classifying which species received the peroxide injections overlap and corroborate those that were significant in the ANOVAs. The convergence of evidence points to the recorded *E. coli* culture responding differentially—here defined as changes in biophoton emission spectra—dependent on what species was actually receiving the injection (Figure 1). These results also reflect phenomena typical of a non‐local or entanglement‐type nature. As expressed by Dotta & Persinger, the occurrence of phenomena that can be classified as *entanglement* or *non‐locality* involves the simultaneous response in one location of space‐time when an event occurs at some other, unconnected location in space‐time (Dotta & Persinger, 2012). Here, we demonstrated that when recording the biophoton emissions of *E. coli* at space‐time Location X, and a second culture of bacteria—*E. coli* or *S. marcescens—*is insulted hydrogen peroxide at space‐time Location Y, the PMT recordings from location X can be used to reliably determine a) whether an event occurred or not, and b) which species was insulted. This is a quintessential definition of “non‐local” interaction, in line with numerous other works (Dotta et al., 2011; Dotta, Mulligan, Hunter, & Persinger, 2009; Persinger et al., 2010).

What is unique about the present study is the nature of the biophoton emissions. The most recent works by Tillbury & Quickenden, Trushin, and even the earliest observations by Gurwitsch himself pointed to a *mitogenetic radiation*, an energy source which influenced and affected the rates of growth of other nearby cells (Gurwitsch, 1923; Gurwitsch & Gurwitsch, 1926; Tilbury & Quickenden, 1988; Trushin, 2003, 2004 ). However, the results of the present study do not directly point to a similar growth‐rate affecting energy. In fact, the responses were recorded when another culture was under extreme stress. Matasushi and colleagues previously demonstrated effects akin to non‐locality when cultures of bacteria are under stress from antibiotics, and in some cases noted the transference of resistance (Matsuhashi et al., 1996). They, and subsequent works, pointed to sound energies as being the carriers of the resistance signals, and noted the ability to influence colony forming rates with the application of particular frequencies, typically in the 10^9^ to 10^12^ Hz (Matsuhashi et al., 1998; Norris & Hyland, 1997).

While it is obvious that *sound* energies are not synonymous with *photon* energies, phenomenologically they reflect the same observation—biological responses in absentia classical biological mediums for signal propagation. It also may be of relevance that the sonic frequency range is identical to the background hydrogen frequency of the Universe (Wouthuysen, 1952). In addition, a number of quantum theories posit the existence of a quasiparticle known as the *phonon*, a quantum field‐equivalent for sonic, or vibrational, atomic interactions first conceived of by the Soviet physicist Igor Tamm (Feinberg & Igor'Evgen'evich Tamm, 1995). Theoretical has since proven to be reality, as the *phonon* is now widely accepted as a quantum mechanical solution to superfluid and condensate equations, and is postulated to be involved with various biological processes (Geesink & Meijer, 2016; Kogar et al., 2017; Scalapino, 2018).

One important piece of information that is required to discern further the nature of the phenomenon reported here are the primary contributing wavelengths of the biophotons. Cosic theorized that every macromolecule can be represented as a biophoton frequency and wavelength, through a process known as the Resonant Recognition Model (Cosic, 1994, 2012 ). In the present study, one might expect to find an increase in biophotons that reflects the upregulation and promotion of alkyl hydroperoxide reductase. Applying the Cosic method to the *ahpC* and *ahpF* subunits suggests wavelengths of 709 and 697 nm, respectively. With respect to works by Tillbury & Quickenden, these are at the extremes of biophoton emissions they recorded as demonstrative of mitogenetic radiation (Tilbury & Quickenden, 1988). We would expect increases in the same wavelength were the phenomenon to be identical; however, additional studies employing filters to determine precisely what wavelength of biophotons were emitted are required before any conclusive statements can be made on the nature of light in the present study. That being said, 700 nm is well within the range of both the measuring capability of our PMT, and what has been reported in previous studies.

Across the levels of biological discourse, a fundamental law exists whereby structure dictates function. While what has been reported thus far is reflective of evidence for non‐local communication through biophotons, there remains the question of *how* this communication occurs; that is, what *structure* enables this *function*. The answer may lie within the genetic code of the bacteria used in this particular study. Taking the number of base pairs for the genomes of both *E. coli* and *S. marcescens* and finding a linear equivalent gives distances of 1564 and 1737 μm (1.564 x 10^−3^ m and 1.737 x 10^−3^ m). It has been demonstrated that the electrons of DNA do not remain stable, but rather are dynamic and move along the backbone of the DNA, at a velocity known as the fine structure velocity, or α. When considered with respect to the speed of light, *c*, the value of α becomes *c*/137. When this velocity (*c*/137) is assumed to be the speed at which electrons move along the DNA backbone of our bacterial species used in this study, frequencies of 1.4 x 10^9 ^Hz for *E. coli* and 1.26 x 10^9^ Hz for *S. marcescens* are computed.

These frequencies are particularly salient as 1.42 x 10^9^ Hz is the background frequency associated with universal molecular hydrogen. Given that many bacterial genomes, including *E. coli*, are circular in nature, this congruence in frequency is even more pertinent given the extensive use of circumscribed electromagnetic field generators in previous experiments (Bazaeal & Helinski, 1968). We have shown that the use of circular magnetic field coils, referred to as toroids, are optimal for producing non‐local effects in a number of experiments when combined with the application of electromotive force (Karbowski, Murugan, & Persinger, 2015; Rouleau & Persinger, 2015; Rouleau, Carniello, & Persinger, 2014). While the present study did not use toroids, the circular genomes are naturally occurring toroids, where the movement of electrons along the genome creates the electromagnetic conditions for non‐local interaction *via* the neutral hydrogen line frequency. We accept this postulate requires additional intensive research to be borne out to fruition. Nevertheless, the overlap between the respective frequencies should not be discounted as mere coincidence.

The nature of biophotons is still at this time subjected to a paucity of knowledge regarding the subject (Fels, 2009). It might be said that there are as many hypotheses for the full causal mechanisms for biophoton interactions as there are individual laboratories conducting research in the field; however, the current study continues in the vein of biophoton research in clearly demonstrating a definite causal link (Rossi, Foletti, Magnani, & Lamponi, 2011). We would like, however, to clarify that it is our claim not the distal group (receiving the peroxide injections) definitively communicated to the proximal group (being measured for biophotons) through biophotons. Our claim is—when a distal bacterial culture is injected with an innocuous substance disrupting the equilibrium of that system, one is then able to measure evidence of that disruption through the biophoton emissions of a separate, proximal bacterial culture. We do not claim the two cultures communicated through light, but rather that by measuring the light emissions of one system we learned information about the other system.

Some argument may also be made with respect to the inherently weak‐nature of biophotons. With such a weak tool for measure, how can one reliably use it as a source of information? Moreover, even environmental sources of light can obfuscate the phenomenon. Although we acknowledge the potential for environmental “spillage” into the measured biophotons, we and other researchers have reliably shown that biophoton measures are incredibly accurate in reflecting their purported systems, with specification down to individual macromolecules and transmitter systems possible (Dotta, Murugan, Karbowski, Lafrenie, & Persinger, 2014). Furthermore, forthcoming publications from completed studies address this exact question. Suffice to say at this time that while there are definite environmental effects on the mean or average biophoton emissions, these effects do not appreciably change the results of statistical tests, and where they do, accounting for environmental influences increases our statistical accuracy, albeit not significantly. Finally, environmental effects do not appear to influence the spectral characteristics of biophoton emissions, only (as stated) the mean or average number of counts recorded. Thus, although the environment was not accounted for in the present study, additional works by the first author and others in the field indicate they would not alter the conclusions of the present study in any meaningful way.

In closing, these results add to the existing bodies of literature indicative of biological systems communicating in absentia classical biological mediums. Given the significant differences from baseline recordings, that the bacteria recorded was never manipulated, and that using the measures of biophoton emissions during the injection of hydrogen peroxide at a distal site, we were able to discern: a) whether an injection had occurred and b) which species received the injection. These results are evidence of *some form* of communication occurring between the two cultures. The precise nature of this communication, aside from being mediated in the electromagnetic spectrum, requires additional study.

## CONFLICT OF INTEREST

None to declare.

## AUTHORS CONTRIBUTION

Manuscript written and reviewed by Lucas Tessaro and Michael Persinger, with data analyses carried out by Lucas Tessaro and Blake Dotta. Experiment was designed and carried out equally by all authors.

## ETHICS STATEMENT

None required.

## DATA ACCESSIBILITY

All data created during this research are openly available from Dryad at https://doi.org/10.5061/dryad.nf1r0f6.
